# Time-constant absolute effect measures for time-to-event outcomes

**DOI:** 10.1186/s12874-025-02743-7

**Published:** 2025-12-17

**Authors:** Oliver Kuss, Annika Hoyer

**Affiliations:** 1 Deutsches Diabetes-Zentrum, Institut für Biometrie und Epidemiologie, Auf’m Hennekamp 65, Düsseldorf, 40225 Germany; 2 Centre for Health and Society, Medical Faculty and University Hospital Düsseldorf, Heinrich Heine University Düsseldorf, Düsseldorf, Germany; 3 German Center for Diabetes Research, Partner Düsseldorf, München-Neuherberg, Germany; 4 Biostatistics and Medical Biometry, Medical School OWL, Bielefeld University, Bielefeld, Germany

**Keywords:** Additive hazard, Survival analysis, Numbers needed to treat, Type 2 diabetes

## Abstract

**Background:**

Reporting treatment effects from clinical trials on both relative and absolute scales is crucial. While absolute measures like the Number Needed to Treat (NNT) are well-established for binary outcomes, their calculation for time-to-event outcomes remains challenging due to time-dependence, which hinders interpretation and communication. Traditional additive hazard models, while addressing time-dependence, have been limited by restrictive assumptions regarding outcome distributions.

**Methods:**

This paper proposes to use a recently introduced class of parametric additive hazard models to compute time-constant absolute effect measures for time-to-event outcomes. These models allow for a wide range of parametric distributions, overcoming the limitations of previous approaches. The approach provides a single, absolute effect size (e.g., hazard difference or NNT) summarizing the effect over the entire study duration. We illustrate this method using digitized Kaplan-Meier data from the EMPA-REG OUTCOME trial, focusing on all-cause mortality, and fit six different parametric distributions (exponential, linear hazard rate, Weibull, log-logistic, Gompertz, and Gamma-Gompertz).

**Results:**

Despite notable differences in model fit across the six distributions, the estimated rate differences, corresponding NNTs, and their confidence intervals were remarkably similar. The linear hazard rate and Gompertz models, which provided the best fit according to the BIC, yielded a rate difference of -8.8 per 1,000 person-years, with an NNT of 114. These models also demonstrated increasing hazards, aligning with expectations for all-cause mortality. The estimated modes of the distributions from the best-fitting models (10.4 and 13.0 years) were more plausible than those from simpler models.

**Conclusions:**

The class of parametric additive hazard models offers a valuable tool for calculating time-constant absolute effect measures for time-to-event outcomes. This approach effectively addresses the issues of time-dependence and limited distribution flexibility, providing a single, interpretable absolute effect size. Future work could explore more general distributions and further derivation of absolute effect measures on the time scale.

## Background

It is generally agreed that treatment effects from clinical trials should be reported on both relative and absolute scales [1, 2]. This is because treatment effects on relative scales often appear more impressive to patients, physicians, and policy makers [3], especially when baseline risks are low.

While the calculation of absolute measures, e.g., the number needed to treat (NNT), is straightforward and well established for binary outcomes, there is less consensus regarding time-to-event outcomes. Here, effect measures are generally time-dependent [4] and must be reported at various time points, hindering accessibility and interpretation. Furthermore, it has been observed, that NNTs for time-to-event outcomes are rarely reported in RCTs. When reported, they are often calculated inappropriately and lack confidence intervals [5].

It was recognized [6, 7] that calculating differences in hazards from additive hazard models effectively addresses the issue of time-dependence. However, an important limitation of traditional additive hazard models is their restrictive assumption regarding the distribution of the time-to-event outcome, where only the exponential and the linear hazard rate distribution [8] had been used [5, 9]. To overcome this limitation while retaining the additive hazard framework, more general models could be employed, such as the initial non-parametric additive hazard model of Aalen [10] or the semi-parametric model of Lin/Ying [11], as suggested by Ke/Jiang [12]. However, utilizing these more flexible models re-introduces the challenge of time-dependence, and also poses difficulties in effectively communicating the results.

Our methodical work reported here was motivated by the need to effectively communicate results from randomized controlled trials (RCTs) in the treatment of type 2 diabetes. In this area, communicating absolute effects is particularly crucial, especially considering that the most influential RCTs of recent decades had been the so-called Cardiovascular outcomes trials (CVOTs) [13]. These trials were mandated by regulatory authorities to investigate patient-relevant outcomes, such as time to a major cardiovascular event or cardiovascular death. These outcomes occurred infrequently in CVOTs (often below 10%), leading to considerable discrepancies between relative and absolute treatment effects, with large treatment effects on the hazard ratio scale appearing far less impressive on an absolute scale [14].

In the following, we propose using our recently introduced class of parametric additive hazard models [15] to compute absolute effect measures for time-to-event outcomes. While these measures still rely on hazard differences, they allow for distributions with essentially arbitrary levels of complexity. Furthermore, they provide a single value summarizing the effect over the entire study time course, finally addressing the three limitations of current approaches: time-dependence, inflexibility of outcome distributions, and reduced communicability.

In the following we introduce our motivating example, re-introduce briefly the class of parametric additive hazard models, and give the results for the example data for six different parametric distributions with varying levels of complexity. A final chapter discusses the strengths and challenges of our approach, gives some additional results, and points to potential future work.

### The motivating example

For illustration, we utilize data from the EMPA-REG OUTCOME trial [16]. This randomized, double-blind, placebo-controlled trial assessed the efficacy of empagliflozin, a sodium glucose cotransporter-2 (SGLT-2) inhibitor, in mitigating cardiovascular morbidity and mortality in patients with type 2 diabetes and elevated cardiovascular risk. The trial involved 7,020 patients observed for a median observation time of 3.1 years across 590 sites in 42 countries. Our analysis here focuses on one of the trial’s secondary outcomes, all-cause mortality. In the treatment group, 267 out of 4687 participants died, whereas 199 out of 2333 died in the placebo group.

Since we lacked access to the original data, we digitized Kaplan-Meier estimates from the original publication using WebPlotDigitizer, version 3.8, an open-source software tool [17]. Subsequently, we extracted the data using the algorithms and R tools of Guyot et al. [18]. The reliability and validity of both methods have been established by us [14, 19, 20] and others [21].

In previous work, we calculated annual NNTs for all-cause mortality in the trial and found them to be 137 [95%-CI: 91, 280], 62 [95%-CI: 41, 122], 39 [95%-CI: 26, 77], and 28 [95%-CI: 19, 56] after 1, 2, 3, and 4 years of treatment, respectively [14]. These values suggest rather modest treatment effects on the absolute scale (e.g., 62 individuals have to be treated with empagliflozin for two years to avoid one additional death), particularly when compared to the hazard ratio for all-cause mortality which was 0.68 [95%-CI: 0.57, 0.82], indicating a relative hazard reduction of 32%.

The full data set is available on the ZENODO repository [22].

## Methods

### The parametric additive hazard model

For a detailed introduction to the model, we refer the reader to our previous paper [15]. In brief, we assume an additive hazard model with the hazard h_x_(t) for an observation with covariate vector x at time t being defined as.

$${\mathrm{h}}_\mathrm{x}\left(\mathrm{t}\right)={\mathrm{h}}_0\left(\mathrm{t}\right)+\mathrm{x}\mathrm\beta,$$

with a parametric baseline hazard function h_0_(t) which is independent of covariates, and a linear predictor xβ which is independent of t. The corresponding probability density function (pdf) is

$$\mathrm{f}_\mathrm{x}=\left(\mathrm{f}_0\left(\mathrm{t}\right)+\mathrm{x}\beta^*\mathrm{S}_0\left(\mathrm{t}\right)\right)\;/\;\mathrm{exp}\left(\mathrm{tx}\beta\right),$$

and the survival function

$$\mathrm{S}_\mathrm{x}\left(\mathrm{t}\right)=\mathrm{S}_0\left(\mathrm{t}\right)\:/\:\mathrm{exp}\left(\mathrm{tx}\beta\right),$$

where f_0_(t), S_0_(t) are the pdf and survival function of the baseline distribution, respectively.

The likelihood function is straightforwardly derived from the standard parametric likelihood function for time-to-event data (see, e.g [23].,, p. 74), with observations with an event contributing the pdf, and censored observations the survival function.

The contribution of a single observation i with covariate vector x_i_ and observation time t_i_ to the log-likelihood function l_i_ is thus

$${\mathrm l}_{\mathrm i}=(1-{\mathrm\delta}_{\mathrm i})^\ast(\log({\mathrm f}_0({\mathrm t}_{\mathrm i})\;+\;\mathrm{x\beta}^\ast{\mathrm S}_0({\mathrm t}_{\mathrm i})-{\mathrm t}_{\mathrm i}{\mathrm x}_{\mathrm i}\mathrm\beta)\;\\_+\;{\mathrm\delta}_{\mathrm i}^\ast(\log({\mathrm S}_0({\mathrm t}_{\mathrm i}))\;-\;{\mathrm t}_{\mathrm i}{\mathrm x}_{\mathrm i}\mathrm\beta),$$

where δ_i_ is the censoring indicator with δ_i_ = 1 if an observation is censored, and δ_i_ = 0 if an event has been observed.

The parameters of the assumed baseline distribution and the β can be conveniently estimated by the maximum likelihood principle. Any software that allows for the coding of a tailored likelihood function, as for example the NLMIXED procedure in SAS or the OPTIM-function in R, can be used for this task.

For practical applications, it is possible to assume a wide range of baseline distributions with varying numbers of parameters. Examples include the exponential distribution with a single parameter, or the linear hazard rate, Gompertz, or Weibull distribution with two parameters. If necessary for the data at hand, even more complex distributions with three or four parameters can be fitted. Model selection criteria, such as the BIC, can be used to compare the results obtained using different baseline distributions.

The primary parameter of interest in our RCT setting is the β coefficient for the treatment effect, which can be interpreted as a hazard difference, and its reciprocal value, the NNT. It can also be instructive to consider transformations of the distribution parameters that have more intuitive interpretations, such as location parameters of the baseline distributions or effect measures that can be interpreted on an absolute time scale.

## Results

**Fig. 1:**
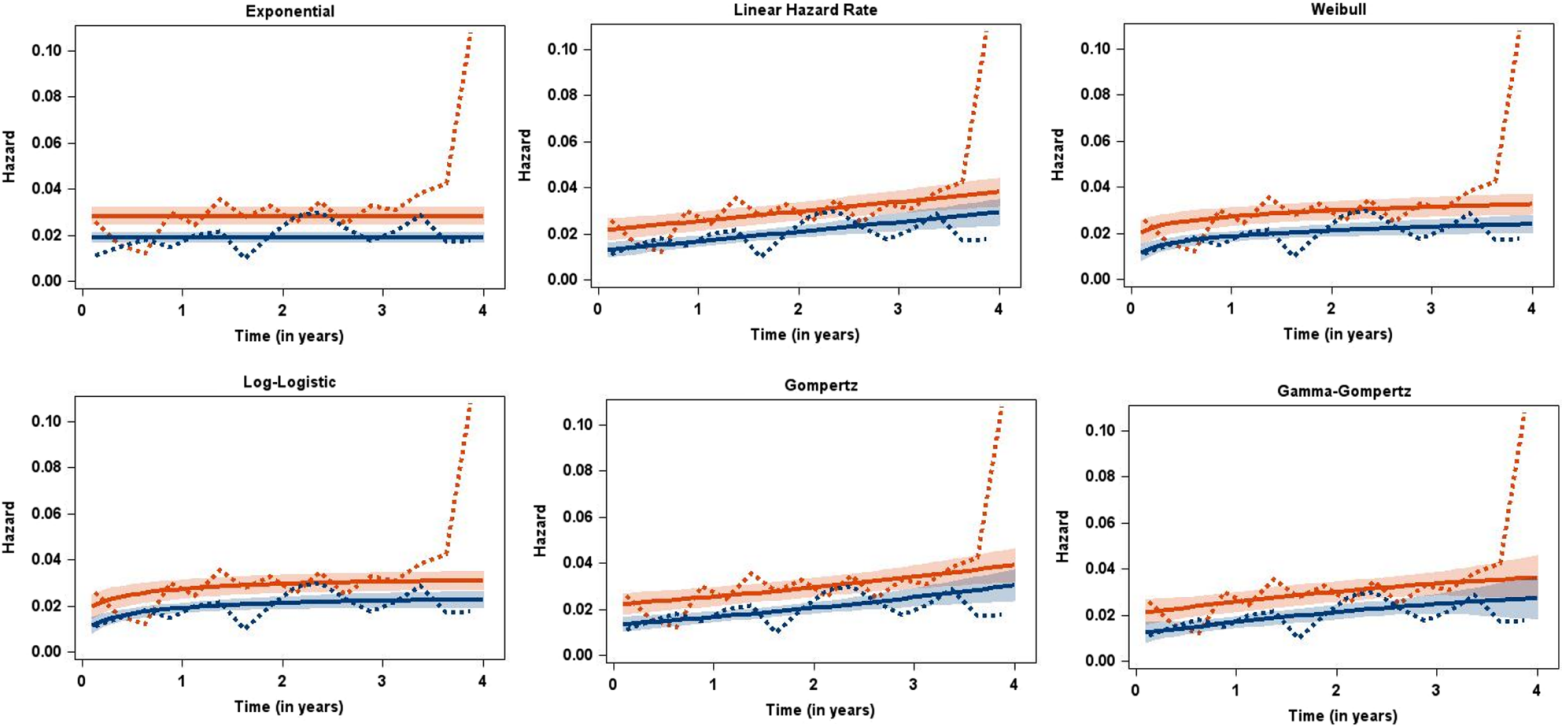
Observed (dotted lines) and estimated (solid lines, with pointwise 95% confidence intervals) hazard functions for the EMPA-REG OUTCOME data (red: Placebo, blue: Empagliflozin) for six different baseline distributions

**Fig. 2:**
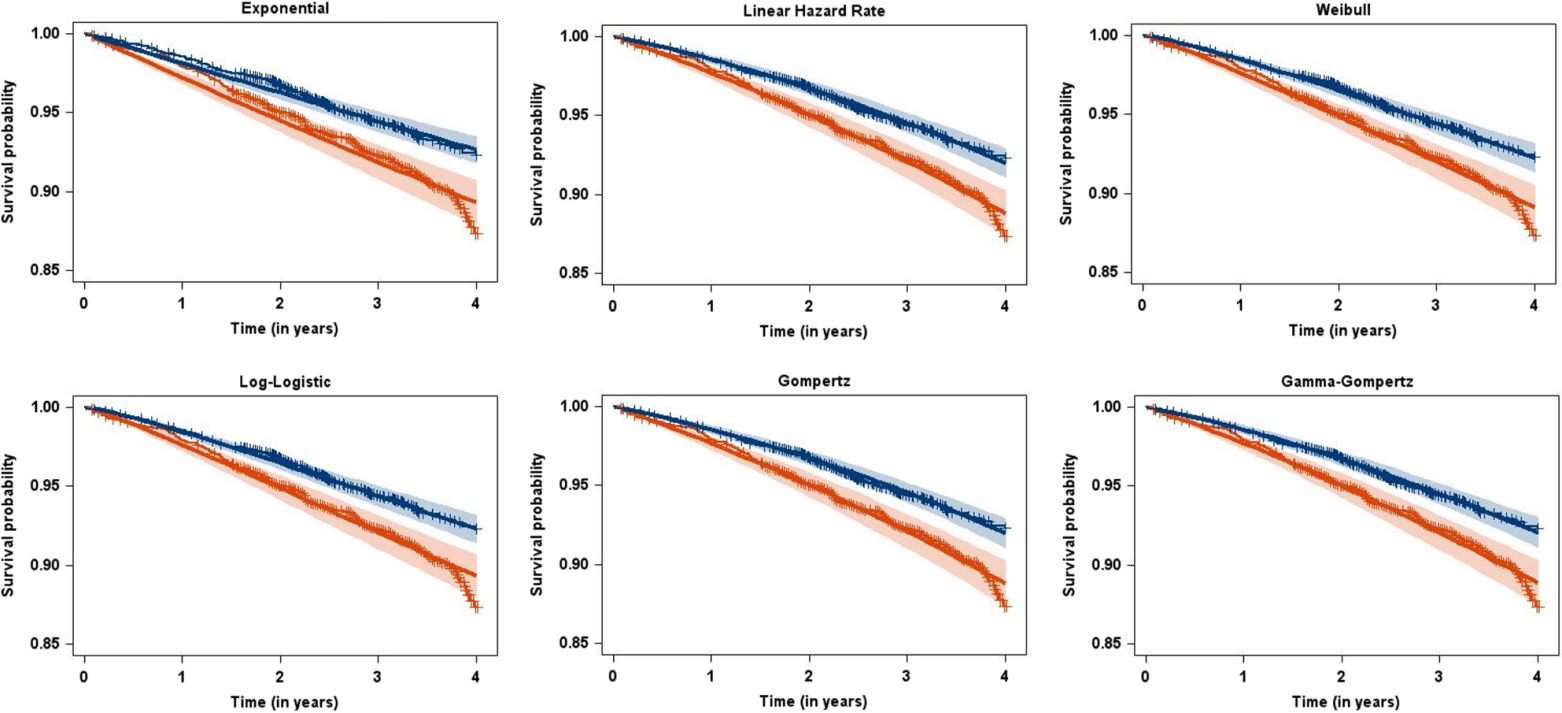
Observed (Kaplan-Meier estimates) and estimated (with pointwise 95% confidence intervals) survival functions for the EMPA-REG OUTCOME data (red: Placebo, blue: Empagliflozin) for six different baseline distributions

**Fig. 3:**
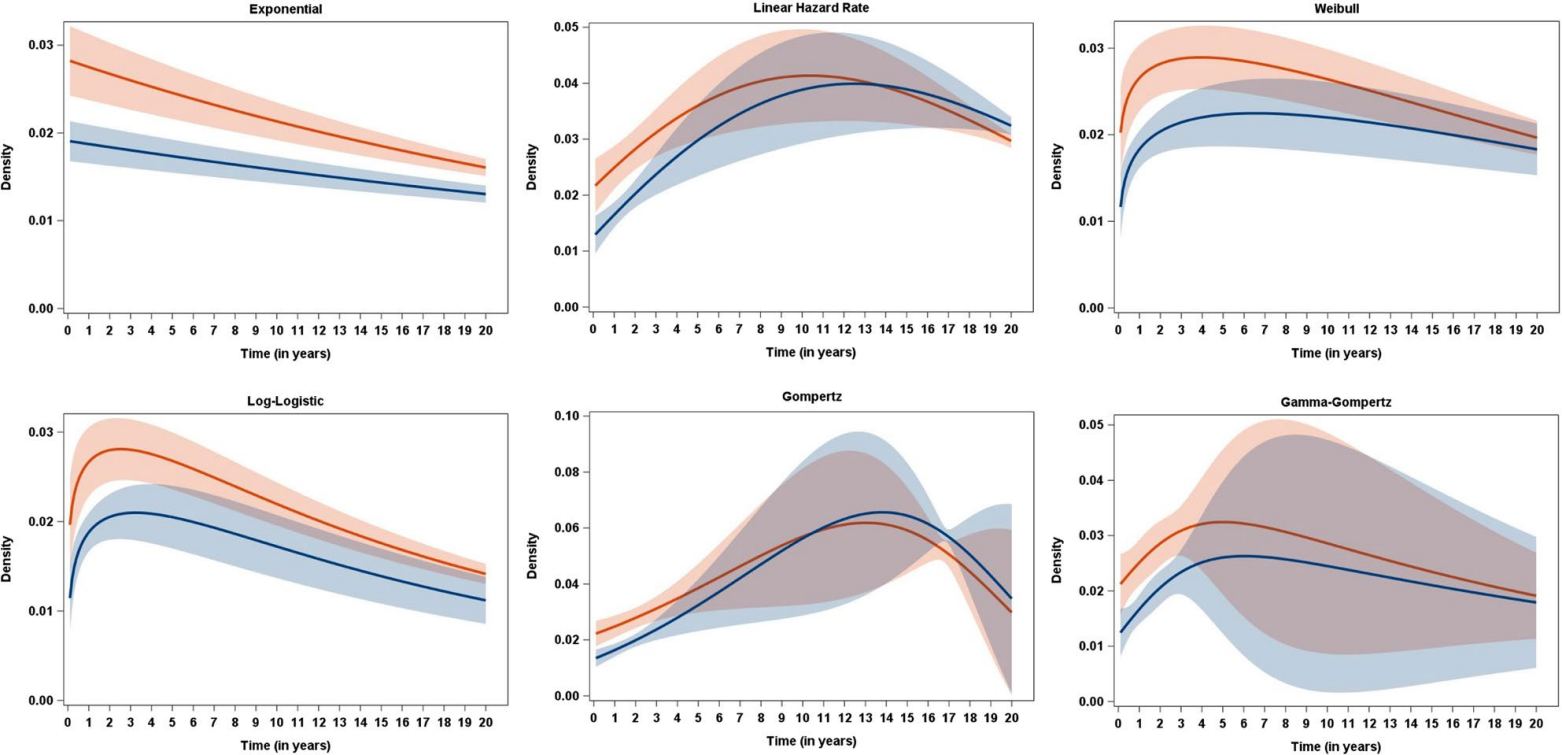
Estimated (with pointwise 95% confidence intervals) density functions for the EMPA-REG OUTCOME data (red: Placebo, blue: Empagliflozin) for six different baseline distributions

Table 1, Figs. 1, 2 and 3 present the results of fitting the parametric additive hazard model with six different baseline distributions to the EMPA-REG OUTCOME data. We began with the simplest distribution, the exponential, which assumes constant hazard functions. For two-parameter distributions, we considered the linear hazard rate (LHR), the Weibull, the log-logistic, and the Gompertz distribution. The LHR distribution is of particular interest as it has been used previously and, while assuming linear hazard functions, allows for both increasing and decreasing hazards, thus generalizing the exponential distribution. The Weibull, log-logistic, and Gompertz distributions are commonly used for mortality data, supported by both empirical [24] as well as mechanistic [25] evidence for their suitability. Finally, we included a three-parameter distribution, the Gamma-Gompertz distribution [26], to assess the necessity of the additional complexity introduced by the third parameter.

**Table 1:** Results from fitting the parametric additive hazard model, the semi-parametric model of Lin/Ying, and models with a piecewise constant hazard function for the EMPA-REG OUTCOME data with the outcome of all-cause mortality

Distribution/Model	Rate difference per 1,000 person-years [95%-CI]	NNT [95%-CI]	Mode [95%-CI]	−2LogL	BIC
One-parametric distribution					
Exponential	−9.2 [−13.8; −4.6]	−109 [−163; −54]	0 (by definition)	4418.7	4436.4
Two-parametric distribution					
Linear Hazard Rate	−8.8 [−13.3; −4.2]	−114 [−173; −55]	10.4 [9.3; 11.5]	4401.8	4428.4
Weibull	−8.6 [−13.2; −4.0]	−116 [−178; −54]	3.9 [2.0; 5.9]	4405.9	4432.5
Log-Logistic	−8.2 [−12.7; −3.7]	−122 [−188; −55]	2.5 [1.4; 3.7]	4407.8	4434.4
Gompertz	−8.8 [−13.3; −4.3]	−114 [−172; −55]	13.0 [10.9; 15.2]	4402.1	4428.7
Three-parametric distribution					
Gamma-Gompertz	−8.8 [−13.3; −4.3]	−114 [−172; −55]	5.0 [−2.7; 12.7]	4401.7	4437.1
Semi-parametric					
Lin/Ying	−9.3 [−13.9; −4.7]	−107 [−212; −72]	--	--	--
Piecewise constant hazard					
5 pieces, 12 months length	−9.0 [−13.6; −4.5]	−111 [−166; −55]	--	4402.0	4455.1
9 pieces, 6 months length	−9.1 [−13.6; −4.6]	−110 [−164; −55]	--	4398.2	4486.8
17 pieces, 3 months length	−8.7 [−13.3; −4.2]	−115 [−174; −55]	--	4381.5	4541.0

Despite notable differences in model fit (as seen from the respective − 2LogL and BIC values), the estimated rate differences, corresponding NNTs, and their confidence intervals are remarkably similar. The LHR and Gompertz models, which give the best fit according to the BIC, yield a rate difference of −8.8 per 1,000 person-years, with a corresponding NNT of 114 and virtually identical confidence intervals. Interpreting the hazard difference, this suggests that treating 1,000 patients with empagliflozin (instead of placebo) for one year could prevent 8.8 events (deaths). The NNT interpretation indicates that 114 patient-years of treatment with empagliflozin are required to prevent one additional death [5, 6].

Figure 1 compares observed and estimated hazards for the six baseline distributions, separated by treatment groups. Observed hazards were calculated from a life-table with quarter-year intervals using the LIFETEST procedure in SAS. Considering the outcome of all-cause mortality and the median observation time of 3.1 years, we anticipated an increasing hazard in both treatment groups, reflecting general secular (and thus increasing) mortality hazards. Indeed, the best-fitting models clearly demonstrate increasing hazards, while the exponential model, assuming constant hazards, provides a poorer fit as measured by the BIC. Interestingly, the models diverge in terms of whether the increase in hazards accelerates or decelerates. The LHR and Gompertz models, both exhibiting the best fit with respect to the BIC, show linearly increasing hazards. This is inherent in the LHR model, while the Gompertz model (characterized by exponentially increasing hazard) achieves near-linearity by adjusting its parameters. Figure 2, comparing Kaplan-Meier estimates from the digitized data to fitted parametric survival curves, corroborates these findings. While the exponential model fit is notably poorer, the fit is generally good for the remaining baseline distributions.

Furthermore, the additional model parameter in the Gamma-Gompertz distribution does not improve the fit. Rate difference estimates and − 2LogL values are virtually identical to those of the corresponding two-parameter distributions, and the fit is not improved when visually examining fitted hazard or survival functions. As expected, the Gamma-Gompertz distribution is penalized by the BIC due to its third parameter, resulting in considerably larger values these indicating a worse fit.

Given the high degree of similarity in estimated absolute effect measures, the substantial differences observed in the respective densities (Fig. 3) are surprising, especially when considering the similar estimated survival functions across baseline distributions in Fig. 2. Examining the modes of the respective baseline distributions (Table 1, Fig. 3) provides an additional plausibility check. The mean age of participants in the placebo group of the EMPA-REG OUTCOME trial was 63.2 years [16, Supplement, p. 48]. Therefore, it is implausible that the time to the most probable age at death (corresponding to the mode of the distribution) would be less than 5 years (or even 0 years as assumed by the exponential distribution). Values estimated from the LHR distribution (10.4 years) and the Gompertz distribution (13.0 years), the two models with the best fit, are by far more plausible.

## Conclusions

In this paper, we demonstrated how our recently introduced class of parametric additive hazard models [15] can be employed to calculate absolute effect measures for time-to-event outcomes. By utilizing additive hazard models with parametric distributions for the outcome, we address the limitations of current approaches, namely their time-dependence, which hinders clear communication, and the restrictions on the flexibility of the outcome distribution. Instead, these models enable the use of outcome distributions with any level of complexity and provide a single, absolute effect size (such as the hazard difference or a NNT). As parametric additive hazard models are standard time-to-event models they naturally allow for censoring and potential variations in follow-up times. Furthermore, they allow for additional covariates and, owing to their parametric likelihood, can be readily extended to handle left truncation, interval censoring, or various other specific characteristics of time-to-event data. Parameter estimation can be realized by maximizing the likelihood function, and, as demonstrated by simulations in our previous paper [15], works well, at least for the situation we envisage here, that of the single binary covariate of treatment in a randomized controlled trial. Further simulations using additional, particularly continuous covariates, which also include violations of model assumptions are an interesting area of future research. Relying on the standard maximum likelihood principle for parameter estimation we also expect unbiased results for more complicated censoring and truncation mechanisms, at least if these can be coded as contributions to the standard parametric likelihood function ([23], p. 74). The same is true for the commonly occurring situation of missing values. In case of missing values that are MCAR („missing completely at random“) or MAR („missing at random“) we also expect unbiased results from parametric additive hazard models, this being guaranteed by the standard maximum likelihood principle. Also standard multiple imputation methods are expected to work well here. Furthermore, in this article we deliberately used the clinical outcome of all-cause mortality to avoid issues with competing risks. For all other clinical outcomes, extensions of parametric additive hazards to deal with competing risks models might be of value.

Relying on parametric distributions for time-to-event outcomes offers an additional advantage: the ability to describe results beyond the models’ generic effect measures. For instance, for the LHR distribution, the difference in modes of the outcome distributions between the treated and control groups has a straightforward form: (−1/b)(-sqrt(b) + 1/sqrt(1/b) + β), where b is the estimate for the common slope of the hazard function in the two groups. For the EMPA-REG OUTCOME data we find an estimated mode difference of 2.1 years [95%-CI: 0.6; 3.6]. This indicates that the most probable age of death is delayed by 2.1 years in the treatment group. A similar derivation can be made for the difference in mean ages of death for the Weibull distribution, and further research could explore deriving additional effect measures in absolute time, such as median differences.

Several options exist for improving and checking model fit. To improve the model fit, one could employ more general models (e.g., the semi-parametric model of Lin/Ying [11], models with piecewise constant hazards, or more general four-parameter distributions). The necessity of this increased complexity should be assessed by referring to model selection criteria that adequately trade off between model complexity and interpretability. In general, and especially for statistical modelling we support Occam’s razor: the principle that, all things being equal, simplicity is preferred over complexity. This is particularly important when choosing parametric distributions for survival analysis, where additional complexity does not necessarily lead to more insights about the data. For example, Cox/Matheson [27] point to the indistinguishability between two three-parameter distributions, the Generalized Gamma and the Exponentiated Weibull distribution, emphasizing that there is essentially no information indicating from which distribution the data were generated. Table 1 gives the results from the semi-parametric model of Lin/Ying [11] and from three models with piecewise constant (PC) hazards where the number and lengths of pieces were varied. All four extended models yield very similar results in terms of effect measures compared to the models with parametric baseline distributions. Due to its construction, the Lin/Ying model does not allow the comparison of the − 2LogL or the BIC to the other models. For the PC models, we find, as expected because of the larger number of parameters, improvement with respect to the value of −2LogL, at least for the models with 9 and 17 pieces. However, these models are heavily penalized by the BIC for their considerably larger number of model parameters. As such, for the EMPA-REG OUTCOME data, the simpler models with two-parametric baseline hazards are clearly chosen as the favored models.

With respect to assessing model assumptions, the situation here - motivated by communicating time-constant absolute effects from a randomized trial with a single binary covariate of treatment - is a rather simple one. We therefore focused only on comparing observed and estimated hazard functions (Fig. 1), and observed and estimated survival probabilities (Fig. 2). For more elaborate additive hazard models (e.g., those including continuous covariates whose non-linearity could be checked), we refer to the ideas of Lefebvre/Giorgi [28]. With the additive model of Aalen et al. [10] in mind they proposed a general strategy for optimal fitting of additive hazard models. We feel that their ideas (checking non-linearity for continuous covariates, graphical displays of residuals) can also inform parametric additive hazard models.

We do not recommend formal statistical goodness-of-fit tests for three reasons. First, these tests are heavily dependent on the sample size. In large samples, trivial deviations from assumptions (here, for example, the additive hazard assumption) might become statistically significant, whereas in small samples large and relevant deviations might remain undetected. Second, such tests are, in general, not specific for the alternative. That is, a rejection of the null hypotheses of additive hazards might also be due to other deviations from the model, such as a non-linearity of continuous covariates. Third, such statistical tests essentially aim to accept the null hypothesis of a good fit which is impossible in the standard setting of statistical tests. Therefore, the testing problem should be instead formulated as an equivalence problem with the null hypotheses describing an irrelevant deviation from the basic assumption.

There has been considerable discussion on the merits of absolute effect measures for time-to-event outcomes [5, 6, 9, 29–31]. All these authors agree that hazard differences must not be interpreted as risk differences and that it is mandatory to communicate absolute effects in units of person-time. Only in rare-event scenarios does the hazard difference approximate the risk difference [5, 30]. Bender and colleagues argued against time-constant absolute effect measures in general [5, 9, 30], citing two primary concerns: (1) the restrictiveness of the additive hazard assumption and (2) the limited availability of suitable distributions for modelling, namely, only the exponential and the LHR distribution. Regarding point (1), this concern is valid, but we should also remember that any model for time-to-event outcomes involves assumptions and we are not aware of any empirical evidence that the additive hazard assumption is less frequently fulfilled than a proportional hazard or an accelerated failure time assumption. Furthermore, model assumptions can be evaluated using the available data. Indeed, we observed very satisfactory model fits under the assumption of additive hazards when comparing the fitted survival functions to the Kaplan-Meier curves (Fig. 2). Regarding point (2) raised by Bender et al., the concept of parametric additive models as introduced here effectively addresses this limitation. By expanding the range of available distributions beyond the exponential and LHR distributions, these models offer greater flexibility and applicability.

There has also been some discussion on whether NNTs for time-to-event outcomes should be named differently to distinguish them from the standard NNTs for binary outcomes. Indeed, terms like “Annualized NNT” (Mayne et al. [7]), “Patient-year adjusted NNT” (Ke/Jiang [12]), or „NYNT“ (Snapinn et al. [32]) have been proposed. Others, e.g. Hildebrandt et al. [5] and Stang et al. [9], however, prefer to retain the familiar term „NNT“. As the latter papers are cited more frequently, we would also prefer to use the term “NNT”. But again, the interpretation of an NNT for a time-to-event outcome is not the number of patients to prevent one additional event, but the number of patient-years. In other words: An NNT for a time-to-event outcome is the inverse of a hazard difference, not of a risk difference. The unit of a hazard (or a hazard difference) is 1/time, whereas risk is dimensionless.

In summary, we think that the class of parametric hazard models is a valuable tool to calculate time-constant absolute effect measures for time-to-event-outcomes. In the future, the model class could be enhanced by further increasing the flexibility of outcome distributions, of course while retaining time-independence. Using the idea of piecewise constant hazards provides a promising avenue for this, and we showed the advantages of this approach recently for the meta-analysis of ROC curves [33].

## Data Availability

The full data set is available on the ZENODO repository: Akbulut C, Kuss O. Data set from "Absolute Treatment Effects for thePrimary Outcome and All-cause Mortality in the Cardiovascular Outcome Trials of New Antidiabetic Drugs – A Meta-Analysis of Digitalized Individual Patient Data. 2022. https://zenodo.org/records/6630421.
